# Nonthermal Atmospheric Pressure Plasma Treatment of Endosteal Implants for Osseointegration and Antimicrobial Efficacy: A Comprehensive Review

**DOI:** 10.3390/bioengineering11040320

**Published:** 2024-03-27

**Authors:** Sogand Schafer, Tina Swain, Marcelo Parra, Blaire V. Slavin, Nicholas A. Mirsky, Vasudev Vivekanand Nayak, Lukasz Witek, Paulo G. Coelho

**Affiliations:** 1 Division of Plastic, Reconstructive and Oral Surgery, Children’s Hospital of Philadelphia, Philadelphia, PA 19104, USA; 2 Department of Biochemistry and Molecular Biology, University of Miami Miller School of Medicine, Miami, FL 33136, USA; 3 Center of Excellence in Morphological and Surgical Studies (CEMyQ), Faculty of Medicine, Universidad de la Frontera, Temuco 4811230, Chile; 4 Department of Comprehensive Adult Dentistry, Faculty of Dentistry, Universidad de la Frontera, Temuco 4811230, Chile; 5 University of Miami Miller School of Medicine, Miami, FL 33136, USA; 6 Biomaterials Division, New York University Dentistry, New York, NY 10010, USA; 7 Department of Biomedical Engineering, New York University Tandon School of Engineering, Brooklyn, NY 11201, USA; 8 Hansjörg Wyss Department of Plastic Surgery, New York University Grossman School of Medicine, New York, NY 10016, USA; 9 DeWitt Daughtry Family Department of Surgery, Division of Plastic Surgery, University of Miami Miller School of Medicine, Miami, FL 33136, USA

**Keywords:** cold plasma, nonthermal plasma, atmospheric pressure plasma, osseointegration, disinfection

## Abstract

The energy state of endosteal implants is dependent on the material, manufacturing technique, cleaning procedure, sterilization method, and surgical manipulation. An implant surface carrying a positive charge renders hydrophilic properties, thereby facilitating the absorption of vital plasma proteins crucial for osteogenic interactions. Techniques to control the surface charge involve processes like oxidation, chemical and topographical adjustments as well as the application of nonthermal plasma (NTP) treatment. NTP at atmospheric pressure and at room temperature can induce chemical and/or physical reactions that enhance wettability through surface energy changes. NTP has thus been used to modify the oxide layer of endosteal implants that interface with adjacent tissue cells and proteins. Results have indicated that if applied prior to implantation, NTP strengthens the interaction with surrounding hard tissue structures during the critical phases of early healing, thereby promoting rapid bone formation. Also, during this time period, NTP has been found to result in enhanced biomechanical fixation. As such, the application of NTP may serve as a practical and reliable method to improve healing outcomes. This review aims to provide an in-depth exploration of the parameters to be considered in the application of NTP on endosteal implants. In addition, the short- and long-term effects of NTP on osseointegration are addressed, as well as recent advances in the utilization of NTP in the treatment of periodontal disease.

## 1. Introduction

Dental implants represent a critical breakthrough in restorative dentistry, serving as a reliable option for the prosthetic replacement of missing teeth. Unlike natural teeth, which are anchored by periodontal ligaments, dental implants rely on osseointegration, a direct structural and functional connection between living bone and the artificial surface of a load-bearing implant. Introduced over 50 years ago by Brånemark et al. in 1969, this concept of integration has evolved from an experimental procedure to a successful and predictable treatment modality, underpinning the growth and development of implant dentistry [1,2]. This is supported by an estimated value of USD 4.99 billion for the global dental implant market in 2023, highlighting the enormous demand for dental implants within restorative dentistry [3]. Yet, the absence of periodontal ligaments in implant prostheses remains a threat in achieving successful osseointegration. Moreover, the presence of an extensive oral microbiota further challenges implant longevity [4]. Current studies indicate that dental implants are prone to bacterial colonization shortly after implantation, with a full spectrum of subgingival flora developing as early as four weeks following implantation [4,5]. In the setting of poor oral hygiene, tobacco smoking, and pro-inflammatory metabolic diseases, amongst many other risk factors, bacterial colonization may often lead to peri-implantitis, an inflammatory condition characterized by alveolar bone loss around the implant [4]. Biofilm formation on implant surfaces by bacteria collectively known as the “red complexes”, including *Porphyromonas gingivalis*, *Aggregatibacter actinomycetemcomitans*, and *Fusobacterium nucleatum*, plays a critical role in the pathogenesis of this condition [4].

To counteract these challenges, significant emphasis has been placed on the development of surface modification techniques to enhance osseointegration and impart antimicrobial properties to implant surfaces. To optimize the interaction with the biological environment, a variety of topographical, chemical, and physical surface modifications have been explored, each seeking to target unique aspects of the titanium implant surface. Chemical and physical surface modifications, including sol–gel [6,7,8], chemical vapor deposition [9], hydrothermal treatments [10], and anodic oxidation [11,12,13], seek to generate functional layers and/or coatings that improve bioactivity and corrosion resistance [8]. Topographical alterations, achieved through processes like sandblasting and acid etching, create textures that mimic natural bone topography, facilitating greater mechanical interlock and cell attachment [14]. Advanced techniques that modify both the surface chemistry and topography, such as a hydroxyapatite coating and anodic spark deposition, have also been employed to enhance bone bonding and osseointegration [15,16].

While these modifications have been found to successfully alter the implant’s surface at a micro- and nano-meter scale to promote bone healing, integration, and a degree of resistance against bacterial adhesion, there remain several concerns [17,18,19,20,21]. For example, significant alterations in surface topography may threaten the bulk structure of the material and potentially compromise the implant’s mechanical integrity and long-term stability [22]. In addition, sterilization methods employed prior to implantation may also negatively alter the treated material. The International Standardization Organization (ISO), a non-governmental global network of national standard bodies which seeks to provide a framework of basic requirements for the manufacturing of medical devices, states within Standard 14937 that sterilization may be achieved through several physical or chemical techniques that achieve appropriate microbicidal activity [23]. While steam autoclave, gamma or electron beam irradiation, and ethyl oxide in a fixed chamber, amongst others, have been deemed as safe and effective Established Category A sterilization techniques by the FDA [5,24,25,26], they may alter the biocompatibility, physical and/or topographical properties of the treated material. This underscores the need for sterilization approaches that preserve functionality and surface characteristics while still effectively achieving the required sterility assurance level (SAL) [27,28,29].

Despite the evolution of surface treatment technologies and exploration of various sterilization methods, the quest for the ideal dental implant—characterized by superior osseointegration, antimicrobial efficacy, and preserved material integrity—remains an ongoing challenge. The interplay between material science, biology, and clinical practices continues to shape the development of dental implants, aiming to address the limitations and enhance the efficacy of these indispensable tools in dental restoration. As the market for dental implants continues to expand and the number of patients requiring such interventions grows, the importance of advancing research in surface modifications and sterilization techniques becomes ever more critical, ensuring long-term success and patient satisfaction with dental implant therapies. The goal of this review is to provide a comprehensive overview of one of these surface treatment technologies, specifically NTP, by looking at the parameters to be considered in the application of NTP on endosteal implants. In addition, we explore the literature for in vitro and in vivo experimentation that have utilized NTP on endosteal implants and discuss their outcomes on osseointegration and antimicrobial efficacy.

## 2. Methods

The selection of articles to discuss NTP application, specifically on endosteal implants, was conducted through a comprehensive search utilizing the PubMed database. The search encompassed a period from January 2000 to December 2023, chosen to capture the nascent development of NTP. Key terms, Medical Subject Headings (MeSH) terms, and Boolean operators (‘AND’ and ‘OR’) were used across each database to refine our search. Search terms included ‘cold atmospheric plasma’, nonthermal plasma’, ‘atmospheric pressure plasma’, ‘bacterial disinfection’, ‘bacterial sterilization’, ‘osseointegration’ and ‘implants’. The search strategy was collectively reviewed by members of the review team prior to execution using the Peer Review of Electronic Search Strategies (PRESS) checklist.

Inclusion criteria for this review encompassed full-text, peer-reviewed research articles that reported primary data on the effects of NTP on endosteal implants, with clear outcomes on osseointegration and/or antimicrobial efficacy. Studies were included if they involved in vitro or in vivo models, with clear methodological descriptions. Exclusion criteria were non-peer-reviewed articles, studies not reporting specific outcomes related to osseointegration or antimicrobial efficacy, and those not utilizing NTP as the primary intervention. Commentaries, editorials, and reviews without original data were also excluded.

**Figure 1:**
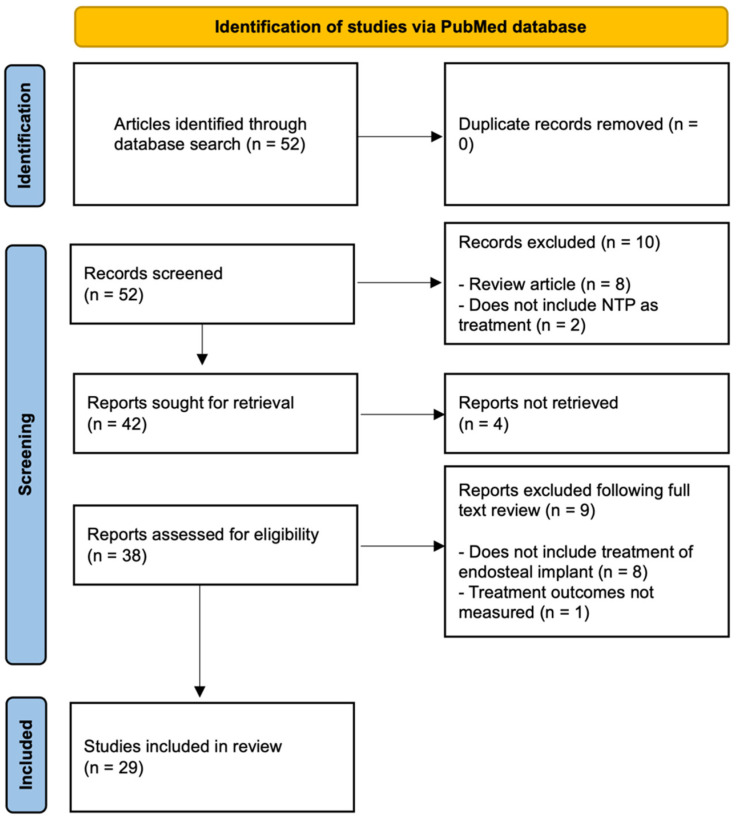
Flow diagram of the literature search.

Fifty-two studies were identified through the database search, of which zero were duplicates. Remaining articles’ titles and abstracts were screened based upon the inclusion and exclusion criteria. Ten of these studies were excluded due to the following reasons: (1) the study did not include NTP as treatment or (2) was a review article. Four of the remaining forty-two studies were not included due to inaccessibility. Following a full-text review of the selected studies, an additional nine studies were excluded: those that (1) did not include treatment of endosteal implants or (2) did not measure relevant treatment outcomes. Ultimately, twenty-nine studies qualified for inclusion and are subsequently elaborated upon (Figure 1).

## 3. Atmospheric Pressure Plasma

**Figure 2:**
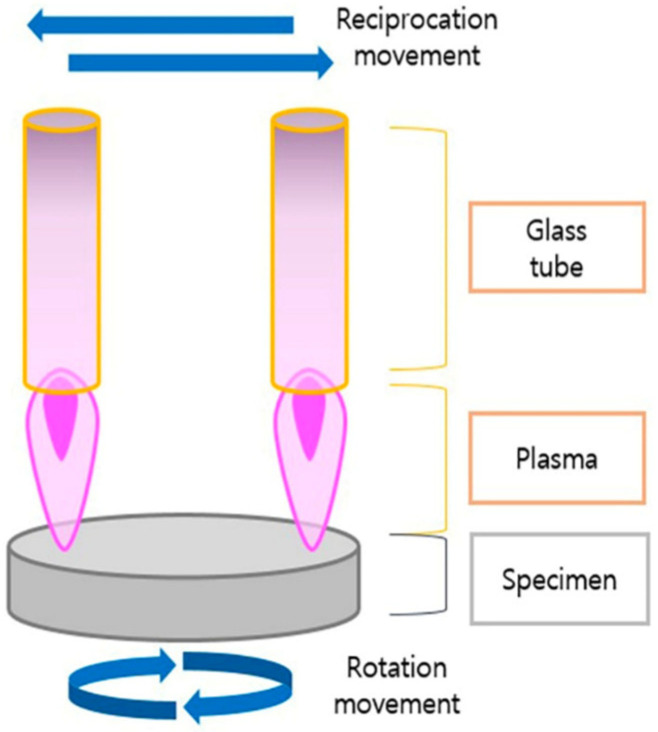
Nonthermal plasma treatment of a specimen. Reprinted from Lee et al. 2022 [4].

Atmospheric pressure plasma, also known as cold atmospheric plasma or NTP, is often described as the fourth state of matter. It consists of ionized gas containing positive and negative ions, electrons, and reactive species, all coexisting in a neutral background gas [5]. NTP is generated at atmospheric pressure and allows for a broad range of surface alterations without the need for high temperatures or vacuum conditions, making it a versatile tool with many biomedical applications. Importantly, the use of NTP has recently been explored as a method to enhance dental implant surfaces [3,5,30,31,32,33,34,35,36,37,38,39] (Figure 2).

Historically, attempts to treat implant surfaces have involved the use of large thermal, radio frequency, and glow discharge plasma devices [34,35]. These earlier technologies either operated at high temperatures or under low pressures and were deemed impractical for on-site clinical use. The inconsistency in equipment operation and concerns over economic viability thereby led to a decline in their use for implant surface treatment. This shift reflected an evolution in the approach to dental implantology, moving away from older, less efficient methods towards more advanced, reliable, and cost-effective solutions provided by NTP systems. NTP’s ability to function near room temperature, as opposed to thermal plasmas which can reach temperatures up to 10,000 K, underscores its suitability for sensitive biomedical applications in the clinical setting [39]. It is well known that there is a strong interaction between new bone formation and the surface characteristics of implants, such as chemical composition, roughness, porosity, and wettability [37,38]. NTP treatment has been reported to significantly increase the surface energy of implants, affecting their hydrophilicity and, consequently, their wettability [36]. This improved wettability has been shown to accelerate bone regeneration by promoting protein and cellular interactions at the implant surface [30,36]. Such interactions have been deemed essential for successful osseointegration at the bone to implant interface, providing a stable foundation for the dental prosthesis [38].

NTP can be generated through various systems, including dielectric barrier discharges, corona discharges, and plasma jets [3,31,33,40]. These systems can be customized with specific gas compositions, like argon mixed with oxygen, to produce reactive oxygen species (ROS) [41]. Such customization abilities facilitate targeted surface modifications, enhancing the implant’s hydrophilicity, biological compatibility, and antibacterial activity [41,42]. Furthermore, NTP possesses the power to polymerize monomers or apply polymer coatings directly onto surfaces, opening new avenues to create bioactive and antimicrobial implant surfaces [43]. The ability of NTP to operate at low ambient temperatures while initiating “high-temperature” chemistry has allowed for surface activation and modification without altering the bulk properties of the implant.

### 3.1. Surface Modifications and Characterization

Characterization techniques such as Scanning Electron Microscopy, Energy Dispersive X-ray Spectroscopy (EDS), X-ray Photoelectron Spectroscopy (XPS), and Fourier-Transform Infrared Spectroscopy (FTIR) are essential tools for analyzing the effects of NTP treatment on dental implants [44,45]. These methods provide detailed insights into the chemical composition, surface energy, and structural changes induced by NTP, facilitating a comprehensive understanding of how these modifications impact the implant’s bioactivity and osseointegration potential [8,41,43,46].

Implant surfaces often accumulate carbon approximately four weeks following production processes, which can lead to the biological aging of titanium [46]. This aging process, which is thought to occur as a result of hydrocarbon presence, renders titanium surfaces hydrophobic. In support of this, previous research has found that 4-week-old titanium surfaces exhibit reduced affinity for protein and osteoblast attachment [47,48]. The presence of hydrocarbon layers on the titanium surface is a significant concern, as it diminishes the surface’s ability to support both osteoblast adhesion and proliferation, which are critical for successful implant integration [48]. Studies which have corroborated the prevalence of adsorbed carbon species on dental implant surfaces have also demonstrated the application of NTP treatment to mitigate this issue [46,49]. More specifically, studies have used XPS to quantitatively determine the chemical composition of the implant surfaces after NTP treatment, revealing a significant reduction in organic contaminants, including carbon, and nitrogen, as well as other elements like fluorides, magnesium, and silicates [41,45,49].

In contrast to hydrophobicity, hydrophilicity has been defined as a critical property of dental implant surfaces as it directly influences the implant’s ability to integrate with bone tissue [50]. A hydrophilic surface promotes better wettability, which enhances the initial protein adsorption and subsequent cellular interactions necessary for bone bonding [20,21,37]. Interpretation of a surface’s contact angle, which assesses surface wettability by a liquid, provides critical insights into surface energy. More specifically, a lower contact angle indicates higher surface energy and enhanced hydrophilicity, which augment initial cellular attachment [4,46,51]. A marked decrease in contact angles with both polar (e.g., distilled water) and nonpolar (e.g., ethylene glycol) liquids has been observed following NTP treatment, indicating a substantial increase in surface energy and hydrophilicity [45]. Improved hydrophilicity after NTP treatment is also a result of reduced hydrocarbon and increased hydroxyl group presence on titanium and zirconia surfaces [49]. An increase in surface oxygen and the formation of thick oxide layers further contributes to greater hydrophilicity and bioactivity of NTP-treated surfaces [49]. These changes have been found to strengthen the chemical interactions between osteoblasts and the implant material, augmenting the stability and longevity of dental implants [37].

### 3.2. The Long-Term Effects of NTP Treatment

Thus far, NTP treatments have been found to improve surface properties, like enhanced hydrophilicity, and optimize chemical composition without compromising the mechanical integrity or altering the bulk properties of the implants [52]. Overall, this ensures that improvements are limited to the surface, which should in turn preserve the implant’s structural strength [52]. While previous studies have explored the potential benefits of NTP treatment in the immediate post-treatment period, it is equally as critical to investigate the longitudinal effects of NTP treatment on dental implants. In this context, several preclinical studies have begun to investigate the long-term effect of NTP treatment.

With respect to the effects of NTP on bone regeneration over time, an in vivo study performed in a rabbit model revealed that plasma-treated implant surfaces led to increased bone formation relative to the control groups (untreated surfaces) in the early and late timepoints of 45- and 90- days, respectively. This highlights NTP’s potential to sustainably enhance bone integration [53]. Furthermore, a preclinical study performed in a pig model demonstrated that titanium implants treated with argon plasma showed significant long-term improvements in bone–implant contact (BIC) values and bone area fraction occupancy (BAFO) over an 8-week period compared to untreated controls [54,55]. With observable benefits extending up to 8 weeks post-treatment, these results suggest that NTP treatment may have an enduring impact on implant stability and integration. Another study performed over 12 months in a mouse model explored the potential carcinogenic impact of NTP on the oral mucosa. The study concluded that repeated NTP exposure did not induce carcinogenic effects or generate lasting non-invasive lesions, suggesting that NTP treatment is safe for long-term use in dental and implant applications and may even support re-osseointegration and wound healing [56]. Collectively, the ability of NTP treatments to achieve long-term enhancements without affecting the implants’ mechanical properties emphasizes their potential in improving patient outcomes within dental implantology [57,58,59].

## 4. In Vitro Studies Using NTPs

### 4.1. NTP Effects on Cell Proliferation and Adhesion

Cell proliferation and adhesion are strongly influenced by surface topography and roughness due to an increase in surface energy. While fibroblasts are more attracted to smooth surfaces, osteoblasts seem to perform better on rough surfaces [60,61,62]. Compared to other surface treatments alone, studies have shown that better results can be achieved using additional NTP treatment (Table 1). For example, Tsujita et al. conducted an analysis on the wettability and osteoblast proliferation on titanium discs (Ti_6_Al_4_V), treated with four different types of coatings: grit blasting, micro-arc oxidation (MAO), titanium plasma spray (TPS), and direct metal fabrication (DMF) [63]. All of these coatings underwent NTP treatment, and the outcomes revealed decreased contact angle across all treated surfaces compared to the untreated controls. Conversely, the cell layer exhibited increased thickness within the plasma-treated samples, and in particular the TPS and DMF groups. Additionally, higher rates of cell proliferation were found in the plasma-treated grit blasting, MAO, TPS, and DMF samples compared to their untreated counterparts.

Exploring non-titanium-based implant materials in comparison to the gold standard, Rabel et al. sought to analyze the response of human osteoblasts and fibroblasts to zirconia and titanium-based implant surfaces treated with nonthermal oxygen plasma [36]. Various surface characteristics, including wettability, cell adhesion, morphogenesis, metabolic activity, and proliferation, were examined. The study revealed that NTP treatment increased the surface wettability of titanium- and zirconia-based implant biomaterials, and this effect was contingent upon the surface topography and initial wettability prior to functionalization. In terms of cell response, plasma functionalization of smooth surfaces impacted the initial morphogenesis of fibroblasts, while osteoblast morphology on rough surfaces was primarily influenced by topography. Despite the differences in cell morphology induced by plasma and topography, the effects were not pronounced enough to elicit a change in cell proliferation behavior. On the other hand, analyzing applications times of NTP treatment, Wagner et al. conducted an evaluation of osteoblast-like cells (MG-63) and human gingival fibroblasts (HGF-1) on zirconia and pure-grade IV titanium discs subjected to varying application periods of NTP [66]. In terms of cell proliferation, their research revealed a significant increase in osteoblast cell proliferation following 60 s of NTP treatment. Moreover, an application time of 120 s resulted in a 1.6-fold increase in cell proliferation. A consistent augmentation in cell proliferation was observed for gingival fibroblasts in both the treated NTP groups. Additionally, their findings indicated that coated titanium surfaces with a calcium-phosphate layer led to a 1.3-fold increase in cell adhesion for MG-63 cells after a 24-h observation period, and a 2.8-fold increase after 48-h.

### 4.2. NTP Effects on Disinfection

Recently, there has been a notable increase in the adoption of plasma sterilization techniques and devices within biomedical applications. The effectiveness of plasma sterilization depends on variables such as gas composition, bacterial strain, and driving frequency, surpassing all alternative non-thermal methodologies. Notably, plasma devices demonstrate a heightened capacity for bacterial eradication compared to conventional methods [70] (summarized in Table 1). The mechanistic foundations of plasma sterilization are intricately linked to various plasma constituents, including ROS, electromagnetic fields, ultraviolet (UV) radiation, ions, and electrons [71]. The eradication of bacteria is facilitated by the effect of hydroxyl radicals on unsaturated fatty acids produced by plasma, resulting in the impairment of membrane lipids [72].

Recent in vitro studies have demonstrated the potential of NTP to serve as a tool to reduce bacterial colonization on dental implants [73,74,75,76]. Kamionka et al. assessed the cleaning efficiency of NTP, air-polishing with glycine, or erythritol containing powders, either alone or in combination with NTP, on subgingival plaque [67]. These treatments were applied to sandblasted/acid-etched and anodized titanium discs at both day-0 and day-5 of incubation after treatment. The findings revealed that the combined approach of air-polishing with NTP yielded the most effective cleaning results compared to individual treatments, a trend that persisted even after day 5. Lee et al. focused on the impact of using NTP jets with helium gas (He-APPJ) to eradicate *Porphyromonas gingivalis* biofilms on sandblasted and acid-etched (SLA) titanium discs titanium discs [3,16,46,77]. Their findings indicated that the bacterial biofilm structure on SLA discs treated with He-APPJ for more than 3 min was effectively destroyed. On the other hand, Ji et al. investigated the inhibition of biofilm formation by subjecting anodized grade IV titanium discs to heat treatments at 400 °C and 600 °C, as well as NTP [69]. Their results demonstrated that the application of plasma to TiO_2_ nanotubes, heat treated at 600 °C, effectively inhibited the adhesion of both *S. mutans* and *P. gingivalis*.

## 5. In Vivo Studies Using NTPs

### 5.1. Preclinical and Clinical Studies on Osseointegration and Disinfection

In vivo studies corroborate previously discussed in vitro studies with respect to improved bone regeneration and bacterial disinfection after NTP treatment, because of the optimization of the dental implant surface [78] (summarized in Table 2). For instance, Jang et al. investigated the effects of NTP on titanium implants in a dog model, revealing that NTP treatment significantly improved BIC and bone volume at 4 weeks compared to controls. Although, it is important to highlight that differences became less significant by 8 weeks, suggesting a superior ability of NTP to enhance early healing outcomes [40]. Similarly, Zhou et al. studied a dog model with peri-implantitis, assessing the adjunctive use of NTP alongside mechanical debridement [79]. The plasma group showed significant improvements in sulcus bleeding index, probing depth, and bone height, with decreased levels of inflammatory markers IL-1β and IL-17, indicating both enhanced bone formation and reduced inflammation. Clinical relevance is further established by Küçük et al., evaluating NTP as an adjunct to non-surgical periodontal treatment in periodontitis patients [80]. The study’s outcomes indicated that NTP application facilitated significant enhancements in “clinical attachment level”, indicative of improved periodontal attachment and reduced periodontal pocket depth. Additionally, it led to a decrease in the “gingival index”, reflecting diminished gingival inflammation, a reduction in “bleeding on probing” rates, signaling decreased gingival bleeding susceptibility, and decreased bacterial counts compared to the control group.

### 5.2. Preclinical and Clinical Studies on Wound Healing

Wound healing encompasses inflammatory, proliferative, and remodeling phases, requiring coordinated cellular activities, including the migration and proliferation of fibroblasts and keratinocytes [83]. Vascularization plays a critical role in wound healing, ensuring oxygen and nutrient delivery for effective tissue repair [83]. Disruptions can result in chronic or nonhealing wounds, such as diabetic foot ulcers and pressure sores, presenting significant medical challenges [84,85]. Effective healing interventions aim to promote essential cellular functions and vascular responses to mitigate these issues. NTP may serve as a viable tool in wound healing given its ability to generate reactive oxygen and nitrogen species, alongside UV radiation and electric fields, without causing thermal damage. The ability to operate at low temperatures while delivering bioactive species is critical to facilitating the healing process, making NTP particularly suitable for wound care purposes.

Preclinical studies show that NTP treatment significantly impacts all stages of the wound healing process. During the initial inflammatory phase of wound healing, NTP treatment was shown to inactivate *methicillin-resistant Staphylococcus aureus*, which are common inhabitants known to prevent healing of chronic wounds [86]. Peroxidative bacterial cell damage, direct mechanical cell lysis, and environmental changes in the wound area (e.g., pH alterations) found after NTP treatment underscore its potential in the context of increasing antibiotic resistance [87,88,89,90]. In the proliferative phase of wound healing, NTP promotes the proliferation and migration of keratinocytes and fibroblasts via enhanced expression of genes related to the synthesis of type I collagen and transforming growth factors (i.e., TGF-β1/2) [91]. Finally, during the remodeling phase, NTP was shown to enhance vascularization through the promotion of endothelial cell activity and growth factor release that improve capillary blood flow and oxygen saturation [92,93].

Clinical investigations further validate the preclinical studies investigating the potential use of NTP as a topical application for wound healing. In a study performed by Pekbağrıyanık et al., forty patients who underwent oral surgery and free gingival graft placement followed by post-surgical NTP treatment or lack thereof were evaluated at days 3 and 7 [94]. Results indicated that the NTP group experienced significantly faster epithelization and better color match, with no notable differences in pain, bleeding, or analgesic drug use, highlighting NTP’s potential to improve oral surgery recovery outcomes. Furthermore, Kisch et al. examined the impact of NTP on cutaneous microcirculation in 20 volunteer patients [95]. The research focused on utilizing laser doppler and photospectrometry to assess how repeated NTP applications affect skin microcirculation. Results showed that NTP significantly increased tissue oxygen saturation and post-capillary venous filling pressure, indicating enhanced blood flow. Importantly, these improvements were found to be sustained after multiple treatments. By improving oxygenation and nutrient supply through enhanced vascularization to an affected area, topical NTP treatment may augment the wound healing process.

## 6. Novel Applications of NTP

Initially recognized for its capacity to modify biomaterials, NTP has emerged as a versatile tool with broad applications. However, recent advancements have unveiled its potential across a spectrum of fields, ranging from regenerative medicine and dermatology to oncology, immunotherapy, and even the food industry. With respect to regenerative medicine, NTP has shown promise in enhancing nerve regeneration [96]. For example, a study performed in a rat model which utilized NTP treatment in transected sciatic nerves found increased Schwann cell density and improved nerve fiber continuity. This indicates NTP’s potential to support the recovery of nerve function [96].

Within the field of dermatology, NTP offers innovative solutions for skin rejuvenation and the treatment of various skin conditions. Studies, such as one conducted by Hadian et al., compared NTP with traditional therapies like long-pulsed Nd:YAG laser for hand rejuvenation, showing significant improvements in skin texture and hydration after NTP treatment [97]. Furthermore, NTP has been explored for its antipruritic effects and its potential in managing psoriasis, offering a new avenue for treating chronic skin diseases by modulating immune responses and reducing inflammation [97].

Oncologic-focused studies have provided evidence that NTP may exhibit remarkable antitumor effects. For example, it has been found to inhibit cell metastasis, induce DNA damage, and promote apoptotic cell death in cancer cells while sparing normal cells [98,99]. This selective cytotoxicity, coupled with the ability to overcome resistance to conventional therapies, positions NTP as a promising cancer treatment strategy. Clinical studies have also demonstrated its efficacy in reducing tumor proliferation and enhancing the immunogenicity of cancer cells, suggesting its role in both direct cancer treatment and immunotherapy [100]. Another novel application that has emerged for NTPs lies in immunotherapy, as it has demonstrated an ability to extend the immunogenicity of vaccines [101]. More specifically, NTP has been shown to increase the expression of immunogenic cell death markers and proinflammatory cytokines, leading to enhanced antitumor immune responses. This has significant implications for developing vaccination strategies against cancer and infectious diseases, including COVID-19, where NTP could offer a novel approach to vaccine development and viral inactivation [101,102].

Finally, NTP treatment has been also extended to the food industry, recognized for its potential to improve food safety and quality [103]. Its application in cold plasma processing aims to extend shelf life, enhance sensory properties, and ensure microbial safety of food products, all while maintaining their nutritional value [98]. This aligns with the growing demand for sustainable and efficient food processing technologies. The novel use of NTP across various disciplines highlights its significant potential to revolutionize treatments in medicine, contribute to food safety, and offer innovative solutions in vaccine development. As research continues, the full scope of NTP applications and its impact on future technological advancements remain promising areas of exploration.

## 7. Conclusions

The integration of surface modifications, particularly through NTP treatments, represents a promising avenue for enhancing osseointegration and ensuring the long-term success of endosteal implants. The versatility of NTP in both surface modification and decontamination underscores its potential as an effective tool in treatments using endosteal implants. Further exploration and standardization of NTP protocols are warranted to optimize its application in diverse clinical scenarios. The combination of innovative surface treatments and effective decontamination strategies holds significant promise for advancing the field of endosteal implantology.

## Figures and Tables

**Table 1:** Efficient NTP treatment—selected in vitro studies.

Cell Proliferation	Material +/− Additional Surface Treatment	Type of Application/Treatment	Control	n/Condition	Type of Cells	Outcome	Reference
	Titanium discs, roughed	NTP (10 s)	Untreated	5	rBMM- SC	Cell adhesion and viability	Lee 2017 [51]
	Titanium discs, SA	NTP	Untreated	N/A	MC3T3	Cell adhesion and viability, proliferation, protein absorption	Patelli 2018 [64]
	Titanium discs, SA	NTP (30 s; 70 s)	Untreated	9	MG-63	Cell spreading	Matthes 2022 [65]
	Titanium discs Zirconia discs	NTP	Untreated	6	HGF-1 MG-63	Cell adhesion, gene expression,	Wagner 2022 [66]
	Titanium discs PEEK discs	O_2_-NTP (120 min) Autoclave (20 min) Gamma-ray irradiation	Untreated	108	MG-63	Cell viability, proliferation, cytotoxicity	Maillet 2023 [5]
**Disinfection**	**Material +/− Additional Surface Treatment**	**Type of application/treatment**	**Control**	**n/Condition**	**Bacterial stain**	**Outcome**	**Reference**
	Titanium discs, mirror-polished	NTP (2 min; 10 min)	Untreated	3	*S. mutants*, *S. aureus*, *K. oxytoca*, *K. pneumonuae*	CFU, biofilm formation, viability, SEM	Lee 2019 [46]
	Titanium discs, anodized, SA	NTP (9 min), APG + NTP(9 min) APE + NTP (9 min)	Untreated, APG, APE,	2	Ex vivo Human biofilm	Biofilm reduction	Kamionka 2022 [67]
	Zirconia discs	NTP (60 s; 300 s; 600 s)	Untreated	N/A	*P. gingivalis*	Bacterial adhesion SEM	Lee 2022 [4]
	Titanium dental implant	NTP (3 min)	Untreated Photodynamic therapy PDT phosphoric acid gel PAG	15	*E. faecalis*	CFU, fluorescent staining	Floerke 2022 [68]
	Titanium discs, anodized, heat treated (600 °C)	NTP (120 s)	Untreated	3	*S. mutans* *P. gingivalis*	Biofilm formation, fluorescent staining	Ji 2023 [69]

Abbreviations: SA: sandblasted/acid etched; MG-63 human osteoblast cell line; N/A: not available; HGF-1: human gingival fibroblast cell line; rBMM-SC: rat bone marrow-derived mesenchymal stem cell; MC3T3: presteoblastic murine cells; APG: air-polishing with glycine; APE: air-polishing with erythritol; CFU: colony forming unit; SEM: Scanning Electron Microscopy.

**Table 2:** Efficient NTP treatment—selected in vivo studies.

Osseointegration	Material +/− Additional Surface Treatment	Type of Application/Treatment	Control	n/Condition	In Vivo Model	Outcome	Reference
	Titanium implant	NTP (60 s)	Untreated	n = 24 total	canine	Surface energy BIC BAFO	Coelho 2012 [55]
	Titanium implant, SA	NTP (10 min)	Untreated	10	canine	BV BIC	Jang 2021 [40]
	Titanium implant	NTP, MD	MD + 2% CHX irrigation	10	canine	SBI PD BH IL-1β, IL-6, IL-17 Peri-implant sulcular fluid	Zhou 2022 [79]
	Titanium implant	NTP	Untreated UV	18	porcine	BIC BAFO	Henningsen 2023 [54]
	Titanium implant	NTP	Untreated	6	canine	Radiographic SEM BIC	Nevins 2023 [81]
**Disinfection**	**Material +/− additional surface treatment**	**Type of application/treatment**	**Control**	**n/Condition**	**In vivo model**	**Outcome**	**Reference**
	Direct NTP treatment to infected tooth	NTP	Standard therapy	25 (internal control	human	CAL PCR ELISA	Küçük 2019 [80]
	Direct NTP to tooth	NTP	Untreated	60 (internal control)	rat	Caries index	Hong et al. 2019 [82]

Abbreviations: BV: bone volume; MD: mechanical debridement; CHX: chlorhexidine; SBI: sulcus bleeding index; PD: probing depth; BH: bone height; CAL: clinical attachment level; PCR: polymerase chain reaction; ELISA: enzyme-linked immunosorbent assay.

## Data Availability

Data are contained within the article.
